# The Clinical Significance of Interstitial Pneumonia with Autoimmune Features in Cryptogenic Organizing Pneumonia: A Prospective Multicenter Observational Study

**DOI:** 10.3390/jcm13226870

**Published:** 2024-11-15

**Authors:** Hisao Higo, Hirohisa Ichikawa, Yukako Arakawa, Yoshihiro Mori, Tomoki Tamura, Shoichi Kuyama, Chiaki Matsumoto, Keisuke Sugimoto, Noboru Hamada, Toshimitsu Suwaki, Junko Itano, Yasushi Tanimoto, Satoru Senoo, Akihiko Taniguchi, Yumi Inukai, Machiko Arita, Satoko Makimoto, Katsuhide Kojima, Takashi Matsushita, Yoshinobu Maeda, Nobuaki Miyahara

**Affiliations:** 1 Department of Allergy and Respiratory Medicine, Okayama University Hospital, 2-5-1 Shikata-cho, Kita-ku, Okayama 700-8558, Japan; prea4jsb@s.okayama-u.ac.jp (H.H.);; 2 Department of Respiratory Medicine, KKR Takamatsu Hospital, 4-18 Tenjinmae, Takamatsu 760-0018, Japan; 3 Department of Respiratory Medicine, National Hospital Organization Iwakuni Clinical Center, 1-1-1 Atago-Cho, Iwakuni 740-8510, Japan; 4 Department of Respiratory Medicine, Japanese Red Cross Kobe Hospital, 1-3-1 Wakihama-kaigandori, Chuo-ku, Kobe 651-0073, Japan; 5 Department of Respiratory Medicine, Okayama City Hospital, 3-20-1 Kitanagaseomote-cho, Kita-ku, Okayama 700-8557, Japan; 6 Department of Allergy and Respiratory Medicine, National Hospital Organization Minami-Okayama Medical Center, 4066 Hayashima, Hayashima-cho, Tsukubo-gun, Okayama 701-0304, Japan; 7 Department of Respiratory Medicine, Fukuyama Medical Center, 4-14-17 Okinogami-cho, Fukuyama 720-8520, Japan; 8 Department of Respiratory Medicine, Kurashiki Central Hospital, 1-1-1 Miwa, Kurashiki, Okayama 710-8602, Japan; 9 Department of Radiology, Okayama University Hospital, 2-5-1 Shikata-cho, Kita-ku, Okayama 700-8558, Japan; 10 Department of Dermatology, Kanazawa University Graduate School of Medical Sciences, 13-1 Takara-machi, Kanazawa 920-8641, Japan; 11 Department of Hematology, Oncology, and Respiratory Medicine, Okayama University Graduate School of Medicine, Dentistry, and Pharmaceutical Sciences, 2-5-1 Shikata-cho, Kita-ku, Okayama 700-8558, Japan; 12 Department of Medical Technology, Okayama University Graduate School of Health Sciences, 2-5-1 Shikata-cho, Kita-ku, Okayama 700-8558, Japan

**Keywords:** interstitial pneumonia with autoimmune features, cryptogenic organizing pneumonia, bronchoalveolar lavage, prospective multicenter observational study, connective tissue disease

## Abstract

**Background:** There are cases of idiopathic interstitial pneumonias (IIPs) that do not meet the diagnostic criteria for connective tissue disease but have clinical features suggestive of autoimmune process. Interstitial pneumonia with autoimmune features (IPAF) was recently proposed as a research concept for these patients. Although several prospective studies on IPAF have been conducted, its clinical significance in cryptogenic organizing pneumonia (COP) remains unclear. **Methods**: Patients aged ≥20 years with suspected COP were prospectively enrolled between June 2018 and December 2022. Among the enrolled patients, those diagnosed with COP based on computed tomography (CT) and bronchoalveolar lavage (BAL) findings were compared between the IPAF and non-IPAF groups. **Results**: A total of 56 patients were enrolled in this study. Of these, 30 were diagnosed with COP and included in the analysis. Clinical and serological features were positive in two and six patients, respectively. Each feature was exclusive, and eight patients (26.7%) were diagnosed with IPAF. There were no differences between the IPAF and non-IPAF groups in terms of clinical features, including BAL findings, laboratory data, CT findings, and clinical course. During the one-year follow-up period, the frequency of COP exacerbation did not differ between the IPAF and non-IPAF groups, and no cases of systemic autoimmune disease or death occurred in either group. **Conclusions**: The COP characteristics of the IPAF and non-IPAF groups are similar in all aspects, and distinguishing between the two groups may be of little significance.

## 1. Introduction

There are cases of idiopathic interstitial pneumonias (IIPs) that do not meet the diagnostic criteria for connective tissue disease, but that have symptoms suggestive of autoimmune process, test positive for autoantibodies, or present histologically with findings suggestive of autoimmune disease [1]. Undifferentiated connective tissue disease was first proposed by Kinder et al. [2], lung-dominant connective tissue disease by Fischer et al. [3], and autoimmune-featured interstitial lung disease by Vij et al. [4]. However, since they all have slightly different diagnostic criteria, in 2015, Fischer et al. reported the concept of interstitial pneumonia with autoimmune features (IPAF) as an official statement of the European Respiratory Society (ERS) and American Thoracic Society (ATS), resulting in the unification of the diagnostic criteria [5]. IPAF is a disease concept that includes multiple types of interstitial pneumonia, including non-specific interstitial pneumonia (NSIP), organizing pneumonia (OP), NSIP with OP overlap, and lymphoid interstitial pneumonia.

A recent prospective observational study on IPAF reported that 70 of the 376 IIP cases (18.6%) met the IPAF criteria, and the IPAF group had a higher frequency of developing collagen disease, fewer acute exacerbations, and a better prognosis [6]. However, more than half of the patients in this study were classified as having unclassifiable idiopathic interstitial pneumonia, and it is unclear whether the same trend exists in each disease group (e.g., NSIP and OP). Therefore, this study focused specifically on cryptogenic organizing pneumonia (COP) and examined whether there are clinical differences in COP between IPAF and non-IPAF groups.

## 2. Materials and Methods

### 2.1. Study Design and Participants

This prospective multicenter observational study was conducted by the Okayama Respiratory Disease Study Group. The participating institutions included Okayama University Hospital, KKR Takamatsu Hospital, National Hospital Organization Iwakuni Medical Center, Japanese Red Cross Kobe Hospital, Kagawa Rosai Hospital, Okayama City Hospital, National Hospital Organization Minami-Okayama Medical Center, National Hospital Organization Fukuyama Medical Center, and Kurashiki Central Hospital. This study was approved by the Institutional Review Board of Okayama University Hospital (no. 1804-050, approval date: 7 May 2018) and all other participating hospitals.

Patients aged ≥ 20 years with suspected COP and scheduled for bronchoalveolar lavage (BAL) or surgical lung biopsy for diagnosing COP were prospectively enrolled between June 2018 and December 2022. Written informed consent was obtained from all the participants. Patients who did not provide consent were excluded from the study. The most reliable diagnosis was made by pathological examination of the lung tissue obtained by surgical lung biopsy. However, surgical lung biopsy is rarely performed for COP because of its good prognosis [7,8,9,10]. Although transbronchial biopsy (TBLB) may be able to diagnose OP [8,11,12], it can sometimes be inadequate due to a small sample size or may misdiagnose the disease due to partial OP of other diseases such as a different idiopathic interstitial pneumonia, vasculitis, or malignancy [7]. Therefore, we established the diagnostic criteria for COP in this study based on computed tomography (CT) images and BAL findings, with reference to a previous study [11]. The diagnostic criteria were as follows: (1) CT shows an OP pattern; (2) microbiological tests using BAL fluid are negative; (3) increase in BAL lymphocyte count (>25%); (4) BAL eosinophil count < 25%; and (5) there is no evidence of a possible cause of secondary OP (e.g., autoimmune disease, history of drug administration, history of radiotherapy). Patients meeting all criteria were diagnosed with COP and included in the analysis. We followed up these patients for one year.

IPAF was diagnosed according to the criteria proposed by the ERS and ATS in 2015, namely at least one positive feature from at least two of the three possible domains (Clinical, Serologic, Morphologic) [5]. In Japan, all the autoantibodies listed in the Serologic domain, except the anti-polymyositis-scleroderma (PM-Scl) antibody, can be measured under the Japanese health insurance system. Therefore, autoantibodies other than the anti-PM-Scl antibody were measured at each institution. Anti-tRNA synthetase antibody levels were measured using the MESACUP™ anti-aminoacyl tRNA synthetase test (Medical & Biological Laboratories Co., Ltd., Tokyo, Japan) detecting anti-Jo-1, anti-PL-7, anti-PL-12, anti-EJ, and anti-KS antibodies [13]. On the other hand, anti-OJ, anti-Zo, and anti-Ha antibodies, which were not included in the MESACUP™ anti-aminoacyl tRNA synthetase test, were not measured. Anti-PM-Scl antibody levels were measured by immunoprecipitation at the Department of Dermatology at Kanazawa University using sera stored at −30 °C at the time of registration.

A central review of the CT images was performed by three readers: two expert radiologists (KK: 20 years of experience and SM: 15 years of experience) and one expert pulmonologist (HH: 16 years of experience). The readers independently assessed whether the images were compatible with the OP. In addition, CT images were evaluated for distribution, predominant pattern, and the presence of a reversed halo sign [14]. All disagreements were resolved by consensus.

### 2.2. Statistical Analysis

Statistical analyses were performed using the EZR software, version 1.36 (Saitama Medical Center, Jichi Medical University, Saitama, Japan) [15]. Binary and continuous variables were evaluated using Fisher’s exact test and the Mann–Whitney U test, respectively. Statistical significance was set at *p* < 0.05.

## 3. Results

### 3.1. Patient Characteristics

**Figure 1:**
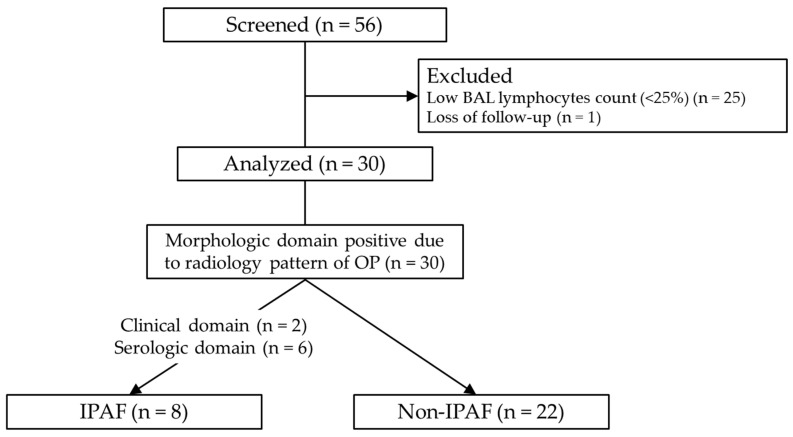
Flow chart of the study. Of the 56 patients, 30 with cryptogenic organizing pneumonia were analyzed. Abbreviations: BAL, bronchoalveolar lavage; OP, organizing pneumonia; IPAF, interstitial pneumonia with autoimmune features.

A total of 56 patients were enrolled in this study. Since no patients underwent surgical lung biopsy, all were screened for COP based on the five criteria described in the Methods Section. Twenty-five patients were excluded because their BAL lymphocyte count was <25% and one patient was excluded because of loss of follow-up. Notably, 30 patients diagnosed with COP were included in the analysis (Figure 1). Regarding the BAL procedure, the volume of saline instilled was 150 mL (n = 20) or 100 mL (n = 10), and the median recovery rate was 46.3% (IQR: 35.5–54.8). Of the 30 cases, 17 were BAL fluid culture-positive, but the isolated bacteria were only endemic oral bacteria such as *alpha-Streptococcus* and *Neisseria*, and no bacteria suspected of causing bacterial pneumonia were detected. All BAL fluid smears and cultures for acid-fast bacilli were negative. TBLB was performed in 15 cases; however, diagnostic specimens were insufficient in eight cases. In the remaining seven cases, pathology findings consistent with OP were observed. The results of IPAF screening are shown in Tables S1–S3 (Supplementary Materials). Two patients had one feature from the clinical domain (distal digital fissure or Raynaud’s phenomenon). Features from the serologic domain were positive in six patients (antinuclear antibody nucleolar pattern, one patient; rheumatoid factor, three patients; anti-topoisomerase, one patient; and anti-tRNA synthetase, one patient). The measurement rate for each antibody was 100% for 7 of the 12 items, and the total measurement rate was 97.5% (351/360) (Table S2). Regarding the morphologic domain, all patients were judged to be positive due to an OP pattern on CT. Four patients had unexplained pleural effusion or thickening, and one had unexplained intrinsic airway disease. Finally, eight patients (26.7%) were diagnosed with IPAF, as all patients met the morphologic domain criteria, and eight patients had one positive feature in the clinical or serologic domain (Figure 1).

### 3.2. Comparison of Clinical Characteristics Between IPAF and Non-IPAF Group

Table 1 shows a comparison between the characteristics of patients with and without IPAF. There were no significant differences in age, sex, body mass index, or smoking history between the two groups. The most frequent symptoms were coughing (70.0%) and dyspnea on exertion (73.3%), with similar frequencies between the two groups. The results of the laboratory examinations are shown in Table 1. The C-reactive protein was similarly elevated in both groups (5.4 mg/dL vs. 5.2 mg/dL), and the serum Krebs von den Lungen-6 was not elevated much in either group (299 U/mL vs. 422 U/mL). We evaluated all patients using BAL, and the total cell count (4.3 × 10^5^ /mL vs. 5.0 × 10^5^ /mL), percentage of lymphocytes (56.5% vs. 60.8%) and CD4/CD8 ratio (0.9 vs. 1.2) were comparable between each group.

**Figure 2:**
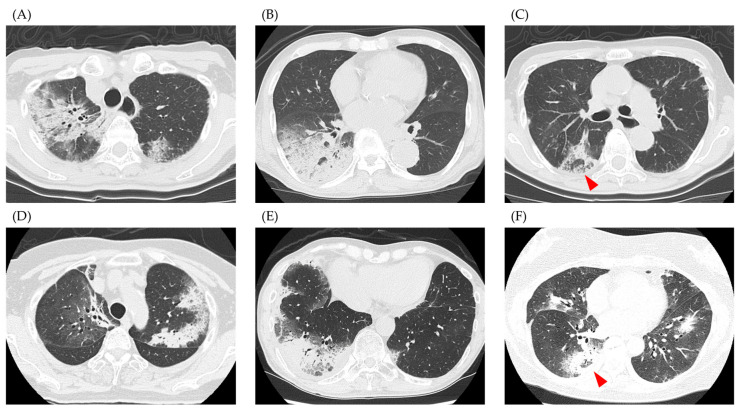
Representative computed tomography images. (**A**–**C**) show cases from the IPAF group, and (**D**–**F**) show cases from the non-IPAF group. (**A**,**B**,**D**,**E**) depict cases with peripheral dominant consolidation, while images (**C**,**F**) show cases with a reversed halo sign (red arrowhead). Abbreviations: IPAF, interstitial pneumonia with autoimmune features.

Representative CT images of the study participants are shown in Figure 2. Figure 2A–C show cases from the IPAF group and Figure 2D–F show cases from the non-IPAF group. Figure 2A,B,D,E show consolidation with a peripheral predominance. The red arrowheads in Figure 2C,F indicate the reversed halo sign. Table 2 shows the results of the central review of CT patterns for both groups. Bilateral and peripherally dominant consolidations were the major patterns observed in our study. A reversed halo sign was observed in four patients. There were no significant differences in the distribution or opacity patterns on CT between the IPAF and non-IPAF groups.

**Figure 3:**
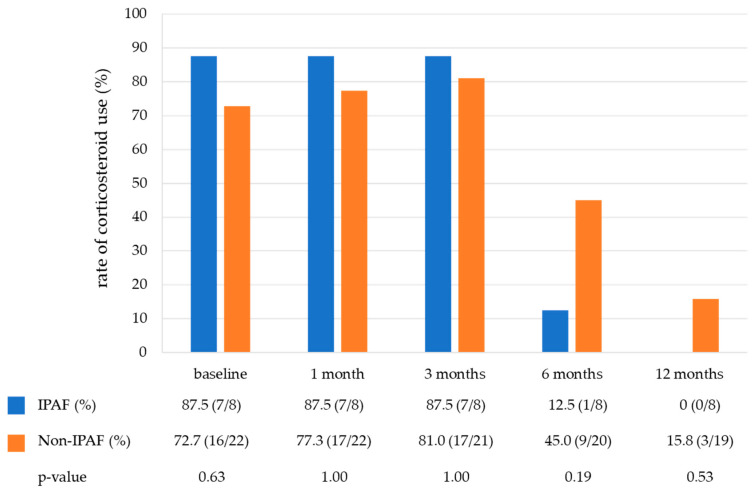
The rate of corticosteroid use in the IPAF group and non-IPAF group during a one-year follow-up period. Abbreviations: IPAF, interstitial pneumonia with autoimmune features.

**Figure 4:**
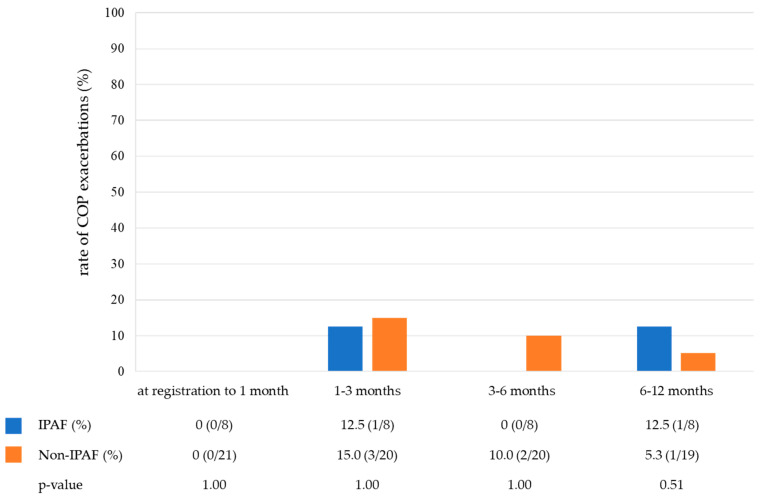
The rate of COP exacerbations in the IPAF group and non-IPAF group during the one-year follow-up period. Abbreviations: COP, cryptogenic organizing pneumonia; IPAF, interstitial pneumonia with autoimmune features.

Table 3 shows the treatment of the COP. Notably, 76.7% of patients (23/30) were treated with corticosteroids, and the starting dose was similar between the two groups (25 mg/day vs. 30 mg/day). None of the patients received immunosuppressive drugs. Figure 3 shows the rate of corticosteroid use in the IPAF and non-IPAF groups during a one-year follow-up period. The rate of corticosteroid tapering was similar between the IPAF and non-IPAF groups. At 12 months, 0% and 15.8% of patients in the IPAF and non-IPAF groups were receiving corticosteroid therapy, respectively, and six patients never received corticosteroids during the observational period (IPAF group, one; non-IPAF group, five). A small number of cases in both groups developed COP exacerbation during the one-year follow-up period. The frequency of COP exacerbation did not differ between the IPAF and non-IPAF groups (Figure 4).

During the one-year follow-up period, no patients developed systemic autoimmune disease or further lung disease in either group. In addition, there were no cases of death in this study.

## 4. Discussion

In this study, we focused on COP among IIPs to determine the frequency and clinical presentation of IPAF. Several prospective observational studies on IPAF have been conducted; however, COP was not their primary population, and IPAF in COP has not been adequately studied [6,16,17,18]. The strengths of this study include a detailed review of clinical features of patients, including BAL, laboratory data, CT findings, clinical course, and high coverage of the serologic domain. In particular, the anti-PM-Scl antibody and anti-melanoma differentiation-associated protein-5 antibody, which have often not been measured in previous reports [6,17,19,20,21,22], were measured in most cases in this study, and the total autoantibody measurement rate was very high at 97.5%.

There are limited data on the frequency of IPAF in COP. A previous prospective study including 21 COP cases reported that 47.8% of the patients met the IPAF criteria [6]. Another retrospective study showed that 17 of the 142 patients with COP (12.0%) met the IPAF criteria [23]. In this study, 26.7% of the patients with COP met the IPAF criteria, suggesting a relatively high frequency of IPAF in COP.

One reason for distinguishing IPAF from IIPs is that the prognosis may differ between the two groups. Among IIPs, the IPAF group has been reported to have a significantly better prognosis than the non-IPAF group in NSIP and unclassifiable IIPs [20,21]. Regarding idiopathic pulmonary fibrosis, several studies have shown a better survival rate in the IPAF group, although the difference was not significant due to the small sample sizes [6,19,20]. Is the IPAF group also associated with a better prognosis in COP, which generally has a good prognosis? Since COP is rarely fatal, the prognosis is expected to be good in both groups. However, is there a difference in response to steroid therapy or in the relapse rate? No deaths occurred in either group, and no differences in steroid tapering or relapse were observed during the 1-year follow-up period.

Another reason for distinguishing IPAF from IIPs is the higher incidence of development of systemic autoimmune diseases [18,24]. Enomoto et al. reported a significantly higher 1-year cumulative incidence of systemic autoimmune diseases in the IPAF group (4.42% vs. 0.89%) [6]. Although it is unclear whether the IPAF group is more likely to develop systemic autoimmune disease with COP, no patients in this study developed these diseases during the 1-year follow-up period, even in the IPAF group. However, considering the reported 1-year cumulative incidence of systemic autoimmune diseases (4.42%/year) [6], the absence of systemic autoimmune diseases in this study may have been due to the insufficient number of cases. Another study reported that 4 of the 20 IPAF patients with an OP pattern on CT developed connective tissue disease [25]. In this report, two of the four patients developed connective tissue disease after more than one year. Therefore, if we had a longer follow-up period, it is possible that our patients would have developed a systemic autoimmune disease. Larger and longer follow-up studies are needed to determine the susceptibility of patients with COP and IPAF to systemic autoimmune diseases.

Our study revealed no differences between the IPAF and non-IPAF groups in terms of clinical background, symptoms, pulmonary function, laboratory examinations, BAL, and CT images as well as the prognosis and incidence of systemic autoimmune disease. Therefore, when treating patients with COP, screening for IPAF using a large number of autoantibody tests may be of limited clinical significance [5].

Our study has several limitations. First, the sample size was relatively small, especially for examining the incidence of developing systemic autoimmune diseases in patients with COP and IPAF. Therefore, larger studies are needed to confirm our results. Second, the diagnosis of COP was essentially based on BAL findings and CT images, with pathological confirmation obtained in only seven cases. Cases diagnosed using surgical lung biopsy should be studied; however, surgical lung biopsy is rarely performed in clinical practice [7,8,9,10]. Relatively high diagnostic accuracy (79%) has been reported with CT alone [26], and BAL has a high positive predictive value of 85% to 90% [11,27]. Because of the high reliability of COP diagnosis by CT and BAL, many studies include cases diagnosed without pathological confirmation [6,25,27,28]. The good treatment response to corticosteroids and favorable prognosis in our cases are consistent with COP [7]. However, the results of this study should only be interpreted as the results of clinically diagnosed COP based on CT and BAL.

## 5. Conclusions

In conclusion, despite having some limitations, we believe that this study is important because no previous study has examined IPAF in COP in as much detail. The characteristics of the IPAF and non-IPAF groups in COP are similar in all aspects, and distinguishing between the two groups may be of little significance. Larger studies are warranted to confirm these results.

## Figures and Tables

**Table 1:** Comparison of patient characteristics between patients with and without IPAF.

	IPAF (n = 8)	Non-IPAF (n = 22)	*p*-Value
Age, yr—median (IQR)	66.5 (59.8–74.8)	68.5 (64.3–75.8)	0.44
Sex (female)—no. (%)	4 (50.0)	5 (22.7)	0.20
Body-mass index—median (IQR)	20.2 (16.9–22.7)	22.4 (18.2–24.3)	0.53
Smoking history			
Former or current—no. (%)	4 (50.0)	14 (63.6)	0.68
Pack-year—median (IQR)	20 (0–58)	12 (0–34)	0.77
Desaturation (≤94%)—no. (%)	1 (12.5)	9 (40.9)	0.21
Cough—no. (%)	6 (75.0)	13 (59.1)	0.67
Sputum—no. (%)	5 (62.5)	7 (31.8)	0.21
Dyspnea at rest—no. (%)	1 (12.5)	6 (27.3)	0.64
Dyspnea on exertion—no. (%)	4 (50.0)	18 (81.8)	0.16
Fever—no. (%)	5 (62.5)	9 (40.9)	0.42
Pathologically proven case—no. (%)	1 (12.5)	6 (27.3)	0.64
Laboratory examinations			
Leukocytes (×10^3^/µL)—median (IQR)	7.5 (6.9–8.5)	7.6 (5.8–10.1)	0.91
Hemoglobin (g/dL)—median (IQR)	12.1 (11.0–12.4)	12.4 (11.1–13.4)	0.50
Platelets (×10^4^/µL)—median (IQR)	39.1 (30.3–42.2)	30.5 (27.4–42.1)	0.57
LDH (U/L)—median (IQR)	182 (173–218)	197 (188–221)	0.56
CRP (mg/dL)—median (IQR)	5.4 (2.2–7.9)	5.2 (0.6–11.1)	0.57
KL-6 (U/mL)—median (IQR)	299 (263–581)	422 (276–792)	0.53
SP-D (ng/mL)—median (IQR)	108 (84.4–294)	161 (118–234)	0.64
SP-A (ng/mL)—median (IQR)	68 (57–74)	74 (46–116)	0.45
Pulmonary function			
FVC (L)—median (IQR)	2.51 (1.87–2.61)	2.63 (2.08–3.04)	0.41
FVC % predicted (%)—median (IQR)	77.0 (68.4–87.6)	79.9 (70.4–93.1)	1.00
Bronchoalveolar lavage			
Total cell count (×10^5^/mL)—median (IQR)	4.3 (3.8–6.2)	5.0 (3.2–6.5)	0.95
Neutrophils (%)—median (IQR)	17.0 (5.6–28.4)	5.1 (2.0–16.8)	0.25
Lymphocytes (%)—median (IQR)	56.5 (41.6–65.5)	60.8 (41.5–66.9)	0.78
Eosinophils (%)—median (IQR)	2.4 (1.0–7.0)	4.5 (1.6–10.6)	0.42
Macrophages (%)—median (IQR)	18.9 (6.0–22.3)	5.3 (3.0–28.0)	0.64
CD4/CD8 ratio—median (IQR)	0.9 (0.5–3.1)	1.2 (0.7–4.0)	0.46

Abbreviations: IPAF, interstitial pneumonia with autoimmune features; IQR, interquartile range; LDH, lactate dehydrogenase; CRP, C-reactive protein; KL-6, Krebs von den Lungen-6; SP-D, surfactant protein D; SP-A, surfactant protein A; FVC, forced vital capacity.

**Table 2:** Computed tomography pattern.

No. (%)	IPAF (n = 8)	Non-IPAF (n = 22)	*p*-Value
Distribution			
Bilateral	7 (88)	17 (77)	1.00
Peripheral dominance/Diffuse	6/2	19/3	0.59
Predominant pattern			
Consolidation/Ground-glass opacity	5/3	19/3	0.30
Reversed halo sign	1 (13)	3 (14)	1.00

Abbreviation: IPAF, interstitial pneumonia with autoimmune features.

**Table 3:** Treatment of organizing pneumonia.

	IPAF (n = 8)	Non-IPAF (n = 22)	*p*-Value
Treated with corticosteroid—no. (%)	7 (87.5)	16 (72.7)	0.64
Starting dose of corticosteroid (mg/day)—median (IQR)	25 (17.5–31.3)	30 (6.3–60.0)	0.34

Abbreviations: IPAF, interstitial pneumonia with autoimmune features; IQR, interquartile range.

## Data Availability

The data presented in this study are available on request from the corresponding author.

## Supplementary Materials

The following supporting information can be downloaded at: https://www.mdpi.com/article/10.3390/jcm13226870/s1, Table S1: clinical domain, Table S2: serologic domain, Table S3: morphologic domain.
