# Supplementary material for: The Clinical Significance of Interstitial Pneumonia with Autoimmune Features in Cryptogenic Organizing Pneumonia: A Prospective Multicenter Observational Study
Source: J Clin Med. 2024 Nov 15;13(22):6870. doi: 10.3390/jcm13226870 (PMC11594663; doi:10.3390/jcm13226870)
Supplement: Supplementary file 1 [file jcm-13-06870-s001.zip › jcm-3293030-supplementary.pdf]

Supplementary Materials

Table S1. Clinical domain (n = 30)

	Positive — no.
Distal digital fissuring	1
Distal digital tip ulceration	0
Inflammatory arthritis or polyarticular morning joint stiffness ⩾60 min	0
Palmar telangiectasia	0
Raynaud's phenomenon	1
Unexplained digital oedema	0
Unexplained fixed rash on the digital extensor surfaces	0
Total	2

Table S2. Serologic domain (n = 30)

	Positive — no.	Measurement rate (%)
ANA ≥ 1:320, or nucleolar or centromere pattern†	1	100 (30/30)
Rheumatoid factor ≥ 2× upper limit of normal	3	97 (29/30)
Anti-CCP	0	100 (30/30)
Anti-dsDNA	0	93 (28/30)
Anti-Ro	0	100 (30/30)
Anti-La	0	100 (30/30)
Anti-ribonucleoprotein	0	97 (29/30)
Anti-Smith	0	97 (29/30)
Anti-topoisomerase	1	100 (30/30)
Anti-tRNA synthetase	1	100 (30/30)
Anti-PM-Scl	0	87 (26/30)
Anti-MDA-5	0	100 (30/30)
Total	6	97.5 (351/360)

† ANA nucleolar pattern in one case. Abbreviations: ANA, antinuclear antibody; CCP, cyclic citrullinated peptide; PM-Scl, polymyositis-scleroderma; MDA-5, melanoma differentiation-associated protein-5.

Table S3. Morphologic domain (n = 30) †

	Positive — no.
Unexplained pleural effusion or thickening	4
Unexplained pericardial effusion or thickening	0
Unexplained intrinsic airways disease	1
Unexplained pulmonary vasculopathy	0
Total (other than radiological and histological patterns)	5

† All patients were judged to be morphological domain positive due to the OP pattern on CT.
